# Does the number of removed axillary lymphnodes in high risk breast cancer patients influence the survival?

**DOI:** 10.1186/s12885-019-5292-2

**Published:** 2019-01-18

**Authors:** Florian Ebner, Achim Wöckel, Lukas Schwentner, Maria Blettner, Wolfgang Janni, Rolf Kreienberg, Manfred Wischnewsky

**Affiliations:** 1 University Ulm, Germany, Prittwitzstraße 43, 89075 Ulm, Germany; 2 HELIOS-Amper Klinikum, Germany, Krankenhausstr. 15, 85221 Dachau, Germany; 3 Department of Gynaecology and Obstetrics, University Würzburg, Germany, Josef-Schneider-Str. 4 · Haus C15, 97080 Würzburg, Germany; 4 Institut für Medizinische Biometrie, Epidemiologieund Informatik (IMBEI), Universität Mainz, Germany, Obere Zahlbacher Straße 69, 55131 Mainz, Germany; 5 Department of Mathematics and Computer Science, University Bremen, Germany, Universitätsallee, 28359 Bremen, Germany

**Keywords:** Advanced breast cancer, Axillary dissection, Lymph nodes, Number of, Sentinel, Survival, Guideline adherent treatment

## Abstract

**Background:**

The decision making process for axillary dissection has changed in recent years for patients with early breast cancer and positive sentinel lymph nodes (LN). The question now arises, what is the optimal surgical treatment for patients with positive axillary LN (pN+). This article tries to answer the following questions:

1. Is there a survival benefit for breast cancer patients with 3 or more positive LN (pN3+) and with more than 10 removed LN?
2. Is there a survival benefit for high risk breast cancer patients (triple negative or Her2 + breast cancer) and with 3 or more positive LN (pN3+) with more than 10 removed LN?
3. In pN + patients is the prognostic value of the lymph node ratio (LNR) of pN+/pN removed impaired if 10 or less LN are removed?

**Methods:**

A retrospective database analysis of the multi center cohort database BRENDA (breast cancer under evidence based guidelines) with data from 9625 patients from 17 breast centers was carried out. Guideline adherence was defined by the 2008 German National consensus guidelines.

**Results:**

2992 out of 9625 patients had histological confirmed positive lymph nodes. The most important factors for survival were intrinsic sub types, tumor size and guideline adherent chemo- and hormonal treatment (and age at diagnosis for overall survival (OAS)). Uni-and multivariable analyses for recurrence free survival (RFS) and OAS showed no significant survival benefit when removing more than 10 lymph nodes even for high-risk patients. The mean and median of LNR were significantly higher in the pN+ patients with ≤10 excised LN compared to patients with > 10 excised LN. LNR was in both, uni-and multivariable, analysis a highly significant prognostic factor for RFS and OAS in both subgroups of pN + patients with less respective more than 10 excised LN. Multivariable COX regression analysis was adjusted by age, tumor size, intrinsic sub types and guideline adherent adjuvant systemic therapy.

**Conclusion:**

The removal of more than 10 LN did not result in a significant survival benefit even in high risk pN + breast cancer patients.

## Background

The nodal status in breast cancer patients is a well-established prognostic factor [1]⁠. In order to evaluate the nodal status surgical staging was routinely done for breast cancer patients with positive lymph nodes. In an axillary dissection the lymphatic tissue was removed between the axillary vein superiorly, the serratus anterior muscle medially and the latissimus dorsi muscle laterally. The number of removed lymph nodes varied from patient to patient. In order to define an adequate removal of lymph nodes a minimum number of 10 nodes in the final pathological report is recommended by the German S3 guidelines [1]⁠. This number has not yet been confirmed by high quality studies, but the morbidity of an adequate axillary dissection is high [2]⁠. Whilst the introduction of the sentinel node biopsy has reduced the morbidity for pN0 patients dramatically the discussion regarding axillary dissection in nodal positive patients is currently ongoing. The publication of studies omitting the axillary dissection in certain subgroups of patients, like the elderly [3]⁠, the results of the Z0011 study and the common application of neoadjuvant chemotherapy, investigating the timing of the sentinel node biopsy and its’ consequences for an axillary dissection [4]⁠ have individualized the surgical treatment. For pN+ patients, surgeons have to choose between axillary dissection (as guideline adherent treatment [1]⁠) and just removing the sentinel [5]⁠. Surgically, the axillary dissection can be done in the above mentioned anatomical structures, but can also be performed as an ‘extended sentinel’ or ‘reduced/non radical axillary’ dissection leaving lymph-fat tissue behind but still removing several lymph nodes. Our previous general analysis on guideline adherent treatment, subgroups and average removed lymph nodes showed no superiority for a higher number of removed lymph nodes [6]⁠.

The clinical question this subgroup analysis investigates is ‘is the removal of 10 and less lymph nodes beneficial compared to the removal of more than 10 lymph nodes’ (as recommended in the S3 guidelines [1]⁠.

In the diverse look at the axillary clearance by current guidelines the tumor biology does not contribute [7, 8]⁠. With HER2+ and triple negative tumor biology patients are considered at a higher risk for recurrence. Systemic treatment is well established in these sub types but the question remains whether more radical surgery also improves the outcome. The current de-escalation in surgery and trial results like the AMAROS trial initiated a discussion of the indication for axillary radiotherapy in patients with breast conserving surgery +/− complete axillary dissection (ALNE) [9]⁠.

As a secondary aim the data was used to retrospectively evaluate the prognostic value of the ratio of positive lymph nodes to removed lymph nodes (LNR).

If the number of removed lymph nodes is reduced the ratio could become less reliable to its mathematical limits.

## Methods

In this retrospective multi-center cohort study, we analyzed data from 2992 patients with primary nodal positive breast cancer, diagnosed or treated at the Department of Gynecology and Obstetrics at the University of Ulm and 16 partner clinics (all certified breast cancer centers) in Germany between 2001 and 2008. The BRENDA (= breast cancer care under evidence-based guidelines) collective contained data on TNM-stage, histological subtype, grading, lymphatic and vascular invasion and estrogen/progesterone/erbB-2-expression. Further details of the database and data collection by medical documentalists have been previously published [6, 10–15]⁠. Certified breast centers are audited once a year to ensure the documentation quality. Date of diagnosis, and all adjuvant therapies (surgery, radiotherapy, adjuvant systemic treatment, endocrine therapy) of all patients meeting the inclusion criteria retrieved for this analysis. Patients gave written informed consent to the collection of their personal data and follow up. Details regarding age, type of surgery and medication used for systemic treatment were also available. Questionnaires were sent to physicians and breast centers involved in follow-up care, to local death registers and to patients to determine the recurrence and survival status of patients. The data was collected by a team of medical documentalists which resulted in high quality data.

Written and informed consent was obtained from all patients included in this clinical study. The inclusion criteria was histologically confirmed invasive breast cancer. The exclusion criteria were carcinoma in situ, primary metastatic disease, bilateral breast cancer, primary occult disease, phylloides tumor and patients with incomplete follow-up.

One of the intentions of the axillary dissection is to reduce axillary recurrences and possibly improve the survival of the patient. The recurrence free survival (RFS) and overall survival (OAS) is also influenced by systemic treatment or radiotherapy. To ensure the quality of the primary treatment the data was checked for 100%-guideline adherence. Wolters et al. demonstrated that treatment recommendations within national guidelines are identical and only differ marginally in adjuvant endocrine therapy [16], therefore guideline adherent treatment was based on the German national consensus S3-guideline [1]. The omission of any suggested adjuvant treatment or the abandonment of any adjuvant treatment was classified as non-compliance with the suggested adjuvant therapy. 100% guideline adherent treatment indicates guideline adherent treatment in all subgroups (operation on the breast, axillary lymph node dissection, chemotherapy (The adjuvant systemic therapy includes patients with neoadjuvant chemotherapy), endocrine therapy, and radiotherapy). The tumor biology was classified according to our previous publication based on the St. Gallen recommendations [6]. The non luminal A cancers were considered ‘high risk’ for further analysis. The classification of variables was age, lymph node ratio (continuous), number of excised lymph-nodes (continuous and 2 categories), hormone receptor (2 categories – with 2 unknown hormone receptor patients included in the hormone receptor positive group), guideline adherent hormonal−/chemo therapy (2 categories), guideline adherent hormonal−/chemo therapy (2 categories) and intrinsic sub types (5 categories).

### Statistical analysis

The primary end points were RFS and OAS. Nominally scaled variables were tabulated in contingency tables and tested for differences in frequency distribution. χ2-tests compared categorical variable between groups. Using a Kaplan-Meier approach standard survival analysis was performed to assess RFS and OAS*.* Formal statistical analysis was done with the log-rank test for the differences between treatment arms with respect to survival (OAS, RFS). The Cox proportional hazards model was used to evaluate simultaneously the effect of several factors on survival and to estimate the hazard ratios (HR) and confidence intervals (CIs). The proportional hazards (PH) assumption was checked using graphical diagnostics based on the scaled Schoenfeld residuals against (transformed) time. Tests were two-sided and a significant level was set at 0.05. The analyses were performed using R version 3.4.2 and SPSS 24.

## Results

Baseline characteristics of the 2992 pN+ patients in this study can be found in Table 1. 2631 (87.9%) patients had more than 10 lymph nodes removed and 361(12.1%) patients ≤10 lymph nodes. The two groups differ significantly for menopausal status, TNM-status, grading, intrinsic sub types and guideline adherent treatment. A detailed distribution of excised versus affected lymph nodes and further tumor details are provided in Table 2. Patients in the subgroup *N* ≤ 10 were older, postmenopausal, had increased occurrence of luminal A tumors with smaller hormone receptor positive tumors with lower grading and fewer infiltrated lymph nodes but were more often not treated according to the guidelines.

**Table 1:**
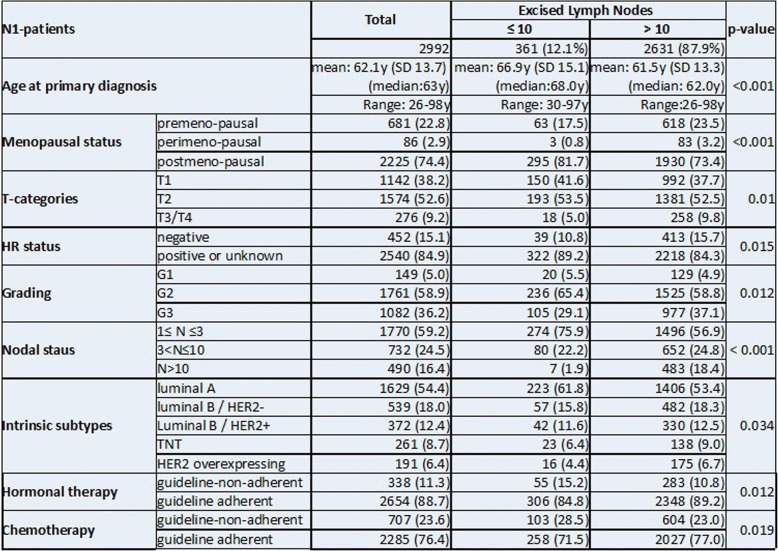
Tumor, patient and treatment characteristics in subgroups

Numbers, percentage and *p*-values, including a cross tabulation of excised LN vs affected LN

**Table 2:** Recurrence free and Overall survival of pN+ patients by number of excised lymph nodes (10 ≤ LN < 10) and adjusted by age, tumor size, intrinsic sub types and guideline adherent adjuvant systemic therapy

	Recurrence free survival				Overall survival			
			95,0% CI for HR				95,0% CI for HR	
	Sig.	HR	Lower	Upper	Sig.	HR	Lower	Upper
Age at primary diagnosis	0.248	1.00	0.99	1.00	< 0.001	1.05	1.04	1.06
Tumor size (mm)	< 0.001	1.01	1.00	1.01	< 0.001	1.01	1.00	1.01
Intrinsic sub types	< 0.001				< 0.001			
Luminal B/HER2-	< 0.001	2.01	1.54	2.61	< 0.001	1.72	1.38	2.15
Luminal B/HER2+	0.003	1.61	1.18	2.21	0.010	1.42	1.09	1.86
TNT	< 0.001	3.55	2.63	4.79	< 0.001	2.96	2.27	3.87
HER2 overexpressing	< 0.001	3.35	2.39	4.70	< 0.001	2.07	1.47	2.91
Guideline-adherent hormonal therapy	< 0.001	1.91	1.43	2.55	< 0.001	2.00	1.59	2.52
Guideline-adherent chemotherapy	< 0.001	1.84	1.46	2.31	< 0.001	1.83	1.51	2.22

The most important parameters for recurrence free survival are intrinsic sub types, tumor size and guideline adherent chemo- and hormonal therapy (Wald statistics)

The Wald statistics showed that the most important factors for RFS and OAS were intrinsic sub types, tumor size and guideline adherent chemo- and hormonal treatment. Age at diagnosis was highly significantly for OAS (*p* < 0.001) but not for RFS. In this multivariable Cox-model the RFS and OAS did not differ significantly for the number of excised lymph nodes (*p* = 0.075 & *p* = 0.376). Further details of the analysis are provided in Table 2. The most important parameters for RFS are intrinsic sub types, tumor size and guideline adherent chemo- and hormonal therapy (Wald statistics). In this multivariable Cox-model neither the number of excised lymph nodes nor age at primary diagnosis are significant (*p* > 0.05).

The most important parameters for OAS are intrinsic sub types, guideline adherent chemo- and hormonal therapy, tumor size and age (Wald statistics). In this multivariable Cox-model the number of excised lymph nodes is not significant (*p* = 0.376).

**Fig. 1:**
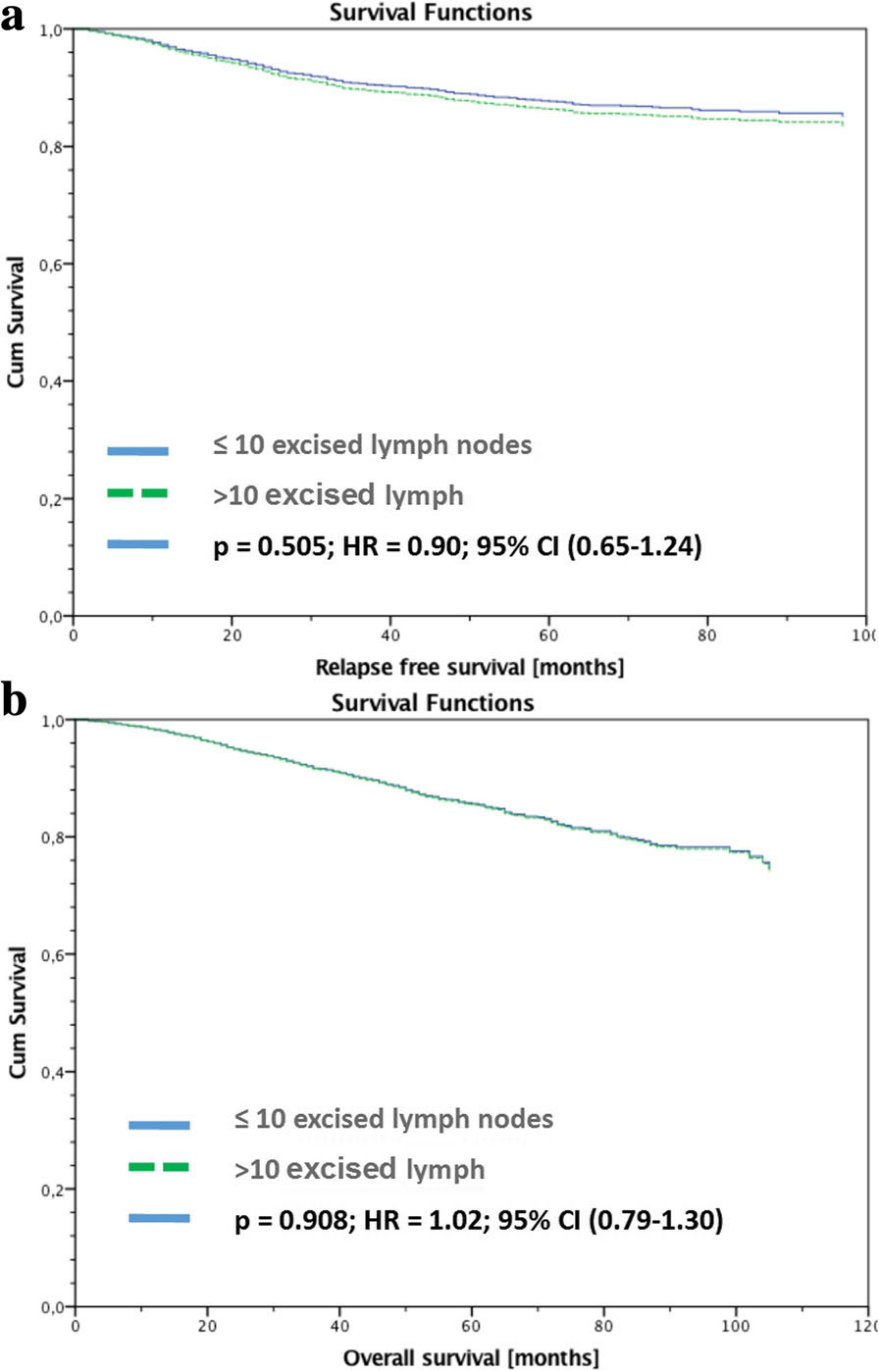
**a**&**b**: Recurrence free (**a**) and Overall survival (**b**) of pN+ patients stratified by number of excised lymph nodes with cut-off value 10 and adjusted by age, tumor size, intrinsic sub types and guideline adherent adjuvant systemic therapy

Figure 1a&b gives the stratified survival function for RFS and OAS. The data was stratified by the number of excised lymph nodes with cut-off value 10 and adjusted by age, tumor size, intrinsic sub types and guideline adherent adjuvant systemic therapy. No significant difference was noted for pN+ patients in this analysis.

As the final step the data was classified into high risk patients (i.e. non-luminal A, Nottingham Prognostic Index ‘high risk’, G3, larger tumor size (T2-T4)). Uni- and multivariable analyses for RFS and OAS for each subgroup again showed no significant survival benefit for removing more than 10 lymph nodes (Table 3).

**Table 3:**
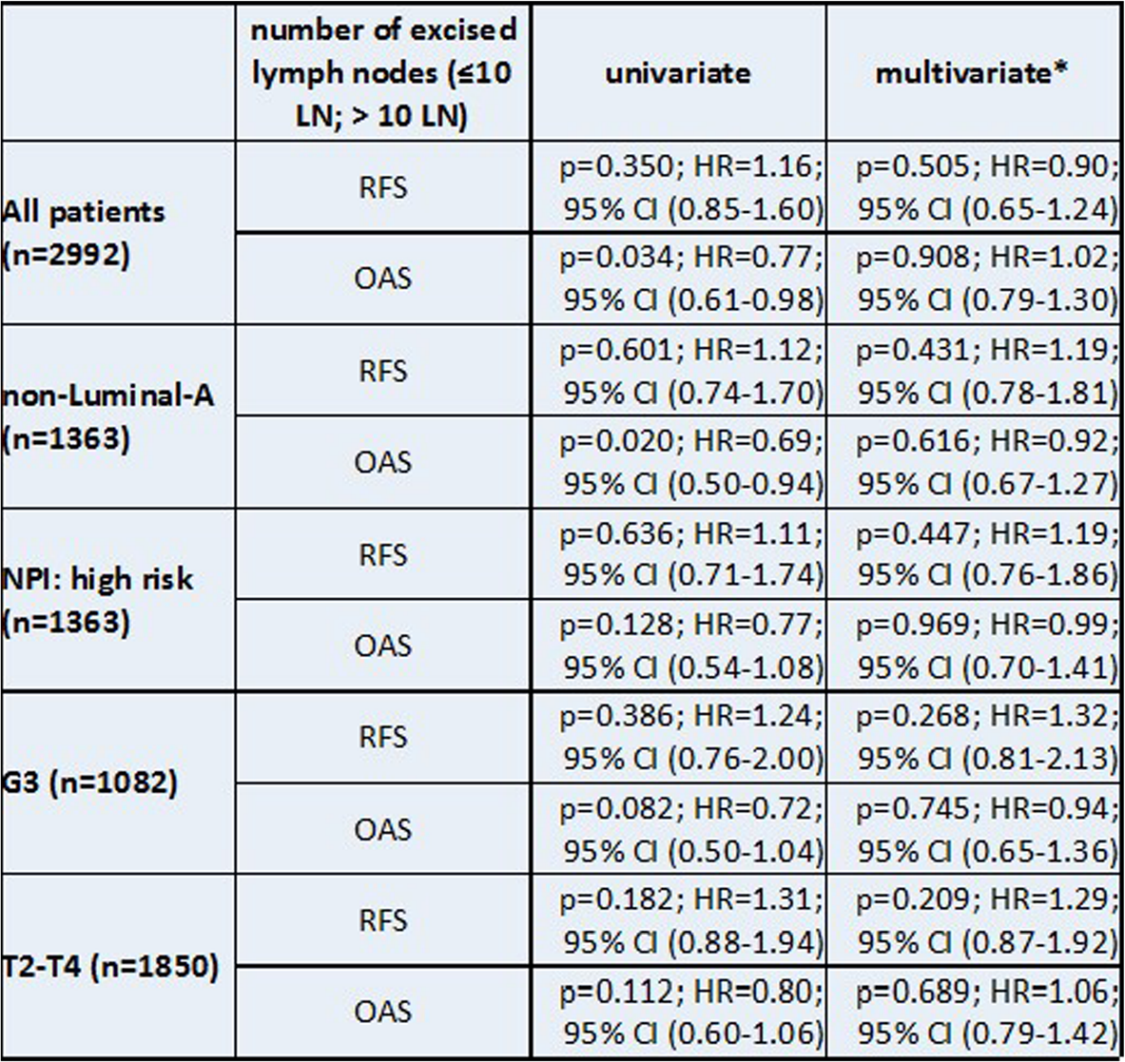
Uni- and multivariable analysis of RFS and OAS with the dichotomized number of excised lymph nodes (10 ≤ LN < 10)

Uni- and multivariable analysis of RFS and OAS with the dichotomized number of excised lymph nodes (10 ≤ LN < 10) for various high risk subgroups of pN1-patients; NPI = Nottingham Prognosis Index; *adjusted by age, tumor size, intrinsic sub types and guideline adherent adjuvant systemic therapy

Next we analyzed the relation between lymph node ratio (LNR) and the number of excised/affected LN and the prognostic value of LNR for RFS and OAS.

**Fig. 2:**
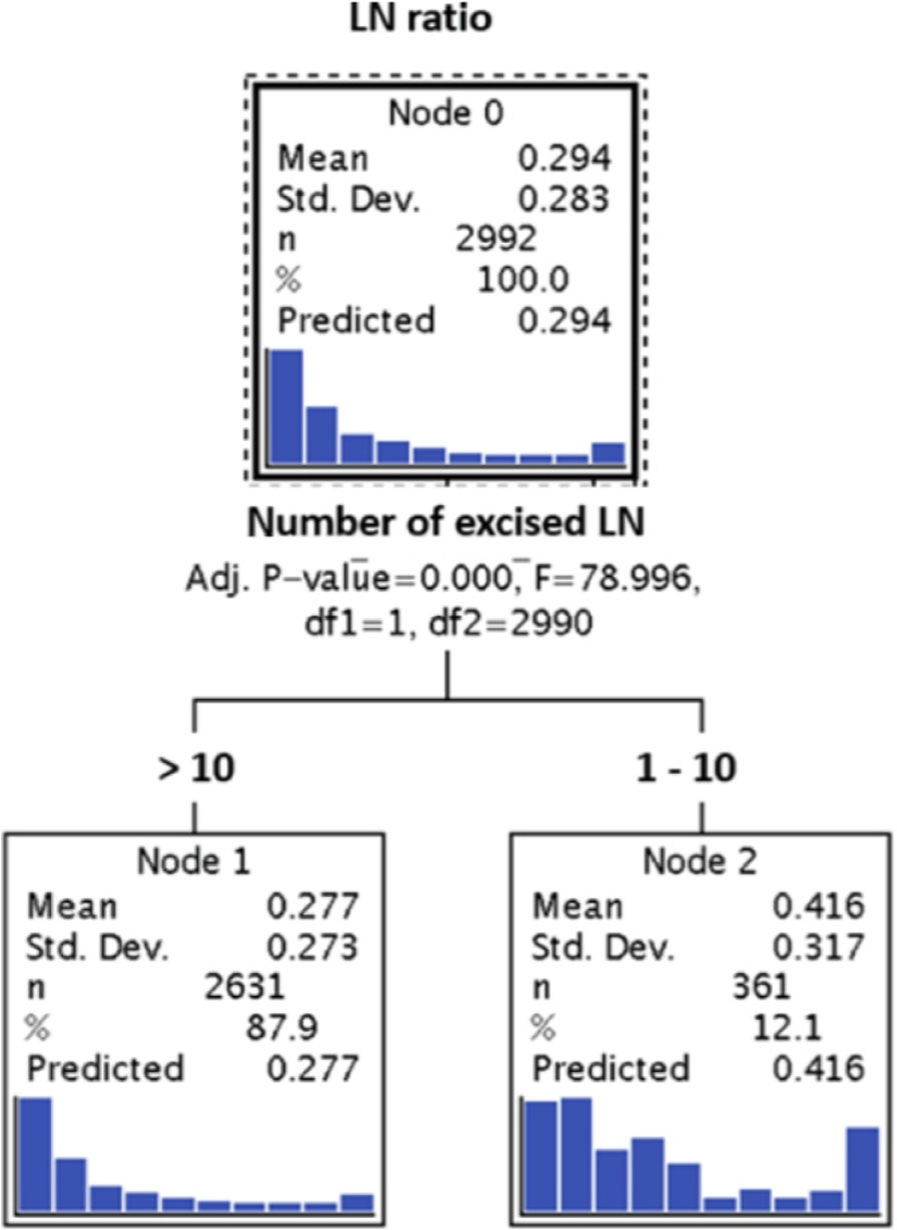
Analysis to determine the ‘optimal’ number of lymph nodes in pN1 breast cancer patients for the calculation of the LN-ratio. Shown here is the calculation with the used cut off of 10 lymph-nodes

**Fig. 3:**
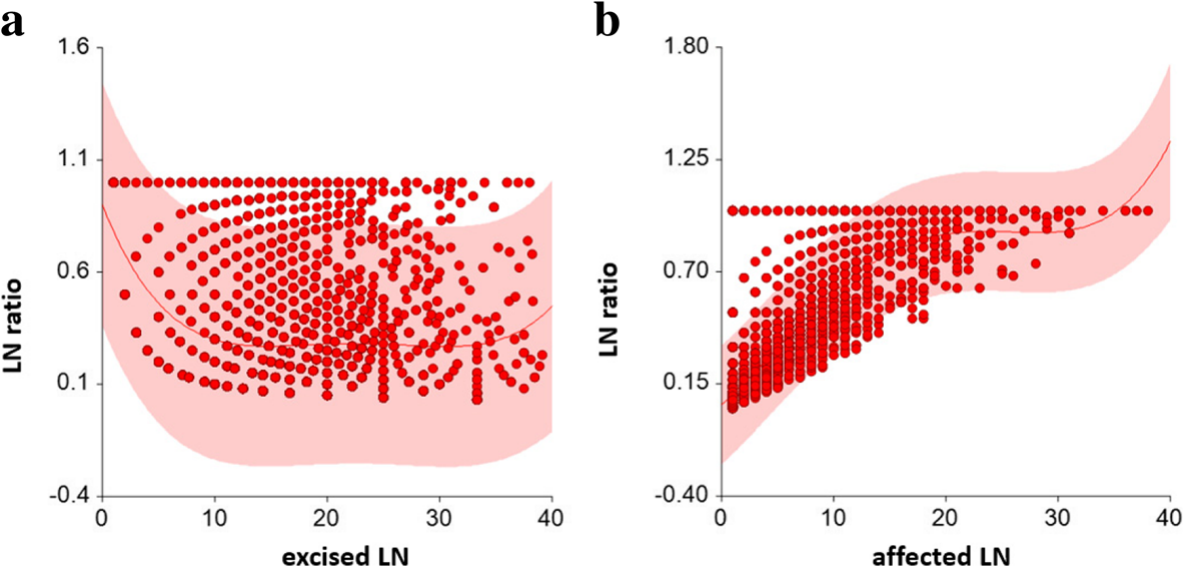
**a**&**b**: Curve fit plot between number of excised LN and LN ratio (3**a**) and number of affected LN and LN ratio (3**b**)

LNR was weakly inversely correlated to the number of removed lymph nodes (ρ = − 0.072; 95% CI: -0.108; − 0.036). Mean (0.42) and median (0.30) of LNR were significantly (*p* < 0.001) higher for pN1 patients with ≤10 excised LN than for patients with > 10 excised LN (mean = 0.28; median = 0.16) (cp. Fig. 2). The curve fit plots (Fig. 3a&b) show that there is no ‘simple’ function between the number of excised (affected) LN and LNR. LNR is uni-variate and multivariable a highly significant prognostic factor for RFS and OAS in both subgroups of pN1-patients with ≤10 and > 10 excised LN (Table 4).

**Table 4:** Uni- and multivariable analysis of RFS and OAS for various high risk subgroups of pN1-patients stratified by LN ratio for pN1-patients with 10 ≤ or more than 10 LN excised

Number of excised LN	LN ratio	univariate	multivariate^a^
≤10	RFS	*P* < 0.001; HR = 5,47; (95% CI 2,30-13,4)	*P* < 0.001; HR = 5,34; (95% CI 2,24-12,70)
	OAS	*P* < 0.001; HR = 4,30; (95% CI 2,28-8,10)	*P* = 0.001; HR = 2,99; (95% CI 1,50-5,56)
> 10	RFS	*P* < 0.001; HR = 6,45; (95% CI 4,74-8,78)	*P* < 0.001; HR = 4,57; (95% CI 3,29-6,36)
	OAS	*P* < 0.001; HR = 6,29; (95% CI 4,77-8,30)	*P* < 0.001; HR = 3,40; (95% CI 2,53-4,56)

Uni- and multivariate analysis of RFS and OAS stratified by LN ratio (as continuous variable) for pN1-patients with 10 and less LN versus more than 10 LN excised; ^a^adjusted by age, tumor size, intrinsic sub types and guideline adherent adjuvant systemic therapy

## Discussion

The results of this retrospective study show no significant survival difference in pN+ breast cancer patients with 10 or less removed lymph nodes compared to more than 10 removed lymph nodes.

The nodal status in breast cancer remains an established prognostic factor alongside the tumor biology [17, 18]⁠. Currently, the surgical standard is the removal of the sentinel node (SN) and until recently, if tumor cells were found in the SN, a ALNE level I&II was performed. Bilmoria et al. published in 2009 a retrospective analysis with a subgroup of ‘macroscopic nodal metastases’ and compared SN biopsy with SN biopsy and axillary completion. The median LN count in the SN only subgroup was 11, so comparable to our subgroup. The relative 5y survival did not differ significantly from the SN biopys and axillary completion group. After adjusting for the clinicopathological features the analysis resulted in a non significant trend for survival and axillary recurrence for the more extended surgery [19]. Looking at the review by Bromham et al. [20] the reviewed literature regarding axillary completion in pN+ patients discussed six trials comparing axillary sampling versus ALNE. The available low-level evidence was considered inconclusive regarding survival and risk of local recurrence. On the other hand, meta-analysis found no significant differences for survival for early breast cancer patients in case of omitting the ALNE. However ALNE is generally considered adequate if at least 10 nodes are retrieved, although this number has been arbitrarily set.

The Z0011-study questioned prospectively the necessity of a systematic/radical level I&II ALNE in patients with up to two positive sentinel lymph nodes. No significant difference in survival was found in the two subgroups. These results are supported by various systematic reviews for this subgroup [5, 21–23] or retrospective studies mainly in elderly or low risk patients [24–29]. The latest update of the NCCN guidelines for breast cancer supports the omission of an ALNE in patients with a T1/2 tumor, maximum 2 positive sentinel lymph nodes with breast conserving surgery and planned whole breast radiotherapy and no preoperative chemotherapy. Parallel to the published results surgeons started to reduce the number of removed lymph nodes, even in patients not fulfilling the inclusion criteria used by the Z0011-study [30, 31].

Some of these results may indicate that even with an advanced axillary finding the ALNE might be less beneficial for the patient [9, 32]. Patients with three or more positive lymph nodes are considered ‘high risk’ for metastasis and decreased survival and have an even worse prognosis if treated sub standard [33, 34]. So for these patients a standard level I&II ALNE is recommended in the current guidelines [7, 8]. With presurgical ultrasound the detection of metastatic lymph nodes is established [35] and may indicate neoadjuvant chemotherapy. If neoadjuvant is not applied, primary surgery does include an ALNE. During surgery metastatic lymph nodes are often palpable but removal of level I-III will increase the post surgical morbidity. Unfortunately the number of removed lymph nodes is often used to define a complete/incomplete axillary resection. With the patients benefit in mind the surgeon has to counterbalance the known long term morbidity of the standard level I&II ALNE versus the questionable oncological benefit of the number of removed lymph nodes [36]. Our data contributes to the discussion about inadequate axillary surgery in pN+ patients as there was no survival difference in the two groups. For the clinician this is a very important information.

Besides the proposed benefit of the surgical removal of tumor cells in lymph nodes the pathological results are used in LNR or logg odds (LODDS) as a prognostic tool. Even with reduced numbers this ratio remains a strong prognostic parameter [18, 24, 37–39]. Here our analysis is in line with the current literature [40] adding knowledge on patients with less than 10 lymph nodes removed.

The number of infiltrated lymph nodes, LODDS or LNR are a criterion for radiotherapy of the axilla. At the German breast expert meeting 2017, it was agreed on axillary radiotherapy for high risk patients with breast conserving surgery and histologically confirmed cancerous lymph nodes (i.e. 1–3 positive lymph nodes and unfavorable tumor biology) or patients with 4 or more positive lymph nodes [41]. Even in patients with just whole breast radiotherapy the lower axillary lymph nodes are included in the treatment [42].This is different in patients with mastectomy. Here further risk factors should exist to justify radiotherapy (i.e. positive SNB, unfavorable tumor biology, young patient). After the publication of the AMAROS trial the indication for axillary radiotherapy is currently discussed [9]. The debate on the benefit of radiotherapy takes into account the different surgeries and histopathological findings.

A current review by Zhao et al. summarizes the available literature for patients with a positive SNB in clinically node negative breast cancer [43]. The authors conclude for this subgroup that the radiotherapy is not inferior to ALNE but this conclusion was only based on 1899 patients with a follow up of 43 or 73 months in two randomized trials [29, 44]. Long term trial results are creating further evidence challenging the need for a surgical ALNE in SN positive, clinically node negative breast cancer patients who receive radiotherapy [9].

Clearly the retrospective data collection of the present study is not designed to answer the question for the optimal number of removed lymph nodes in pN+ patients. Retrospective studies may only allow us to draw associations between the number of removed lymph nodes, guideline-adherent treatment, tumor biology and survival parameters. To draw causal conclusions concerning survival parameters would only be appropriate if treatment allocations were randomized and prospective. However, a randomization regarding the number of removed/affected lymph nodes is hardly possible. Therefore retrospective data might be the only possibility to reducing the morbidity of ALNE without compromising the patients’ long term safety.

The comprehensive BRENDA database with a large number of patients and a long follow up provided by a network of 17 certified breast cancer centers with information on treatment and survival, annual audits for the data quality has provided valuable data for healthcare research [6, 10, 11, 13–16, 45]. The follow up was done according to the German breast cancer guidelines [1]. This includes a clinical follow up every three months in the first two years and then twice a year until year five and then annually.

Similar results have been published in the past but the study populations differed (or less details were provided by the authors) from our study population regarding the nodal status, total number of lymph nodes removed, guideline adherence of the treatment, follow up period, patient numbers and tumor information. For example in our study only patients with axillary metastasis were included compared to 23% node positive in Sanghanis review [21]. Zhang et al. and Liang et al. only reviewed studies including patients who were 60 years or older [22] or considered elderly [46]. To the best of our knowledge there is no literature on the ‘optimal’ number of removed lymph nodes in our focus group. Joyce et al. [47] reports on patients without an ALNE to have a significantly higher risk of axillary recurrence and a slightly elevated mortality rate. Liang et al. confirm the raised local recurrence rates in elderly cN0 patients but do not find an OAS effect. The main difference to the previously mentioned reviews was the inclusion date for the search. Neither review provided information on further treatment even though systemic treatment influences the local disease control. All authors recommend to proceed with ALNE unless there is further evidence available. Our data supports the need for a critical review of the extend of the axillary dissection taking into account the evidence for the oncological benefit and the long-term morbidity.

Our study does not query the need for an ALNE in patients with node positive breast cancer, but it does try to bridge the gap between level I&II ALNE and SNB by looking at the number of removed lymph nodes. A lower number of removed lymph nodes should result in fewer surgical side effects like seroma [48] and ultimately improving the quality of life for the patient without compromising the oncological safety.

To the best of our knowledge this is the first time the number of removed lymph nodes in pN+ patients has been investigated in non-luminal A breast cancer in such detail, number of cases and the effect it has on survival.

## Conclusion

Considering all these facts, our data suggests that removing more than 10 lymph nodes appears to provide no benefit in the survival of the pN+ breast cancer patient.

## References

[1] Kreienberg R, Kopp I, Albert U, Al E. Interdisciplinary S3 guideline for diagnosis and therapy of breast cancer in women. Ger Cancer Soc. 2008.

[2] Soares EWS, Nagai HM, Bredt LC, da Cunha AD, Andrade RJ, Soares GVS (2014). Morbidity after conventional dissection of axillary lymph nodes in breast cancer patients. World J Surg Oncol..

[3] Javid SH, He H, Korde LA, Flum DR, Anderson BO (2014). Predictors and outcomes of completion axillary node dissection among older breast cancer patients. Ann Surg Oncol.

[4] Glechner A, Wöckel A, Gartlehner G, Thaler K, Strobelberger M, Griebler U (2013). Sentinel lymph node dissection only versus complete axillary lymph node dissection in early invasive breast cancer: a systematic review and meta-analysis. Eur J Cancer..

[5] Janni W, Kühn T, Schwentner L, Kreienberg R, Fehm T, Wöckel A (2014). Sentinel node biopsy and axillary dissection in breast cancer: the evidence and its limits. Dtsch Arztebl Int.

[6] Ebner F, Wöckel A, Janni W, Kreienberg R, Schwentner L, Wischnewsky M (2017). Personalized axillary dissection: the number of excised lymph nodes of nodal-positive breast cancer patients has no significant impact on relapse-free and overall survival. J Cancer Res Clin Oncol..

[7] NCCN. NCCN clinical practice guidelines in oncology (NCCN guidelines ® ) breast Cancer version 2.2018 [internet] (2018).

[8] Leitlinienprogramm der (Deutsche Krebsgesellschaft; Deutsche Krebshilfe; AWMF) (2018). S3 - Leitlinie Früherkennung, Diagnose, Therapie und Nachsorge des Mammakarzinoms. AWMF Registernummer: 032 - 045OL.

[9] Yan M, Abdi MA, Falkson C (2018). Axillary Management in Breast Cancer Patients: a comprehensive review of the key trials. Clin Breast Cancer..

[10] Ebner F, Hancke K, Blettner M, Schwentner L, Wöckel A, Kreienberg R (2015). Aggressive intrinsic subtypes in breast cancer: a predictor of guideline adherence in older patients with breast cancer?. Clin Breast Cancer..

[11] Wolters R, Ebner F, Janni W, Novopashenny I, Wöckel A, Kreienberg R (2016). Do T1a breast cancers profit from adjuvant systemic therapy? A multicenter retrospective cohort study of 325 T1a-patients. Arch Gynecol Obstet.

[12] Schwentner L, Wöckel A, König J, Janni W, Ebner F, Blettner M (2013). Adherence to treatment guidelines and survival in triple-negative breast cancer: a retrospective multi-center cohort study with 9,156 patients. BMC Cancer..

[13] Wöckel A, Kurzeder C, Geyer V, Novasphenny I, Wolters R, Wischnewsky M (2010). Effects of guideline adherence in primary breast cancer-a 5-year multi-center cohort study of 3976 patients. Breast..

[14] Schwentner L, Dayan D, Wöckel A, Janni W, Kreienberg R, Blettner M (2018). Is extracapsular nodal extension in sentinel nodes a predictor for nonsentinel metastasis and is there an impact on survival parameters?-a retrospective single center cohort study with 324 patients. Breast J..

[15] Wöckel A, Wolters R, Wiegel T, Novopashenny I, Janni W, Kreienberg R (2014). The impact of adjuvant radiotherapy on the survival of primary breast cancer patients: a retrospective multicenter cohort study of 8935 subjects. Ann Oncol.

[16] Wolters R, Regierer AC, Schwentner L, Geyer V, Possinger K, Kreienberg R (2012). A comparison of international breast cancer guidelines - do the national guidelines differ in treatment recommendations?. Eur J Cancer.

[17] Liao G-S, Chou Y-C, Hsu H-M, Dai M-S, Yu J-C (2015). The prognostic value of lymph node status among breast cancer subtypes. Am J Surg.

[18] Chang Y-J, Chung K-P, Chen L-J, Chang Y-J (2015). Ratio and log odds of positive lymph nodes in breast Cancer patients with mastectomy. Surg Oncol.

[19] Bilimoria KY, Bentrem DJ, Hansen NM, Bethke KP, Rademaker AW, Ko CY (2009). Comparison of sentinel lymph node biopsy alone and completion axillary lymph node dissection for node-positive breast cancer. J Clin Oncol.

[20] Bromham N, Astin M, Hasler E, Mw R, Bromham N, Schmidt-hansen M (2017). Axillary treatment for operable primary breast cancer ( review ) axillary treatment for operable primary breast cancer. Cochrane database Syst rev..

[21] Sanghani M, Balk EM, Cady B (2009). Impact of axillary lymph node dissection on breast cancer outcome in clinically node negative patients: a systematic review and meta-analysis. Cancer.

[22] Zhang P-Z, Chong L, Zhao Y, Gu J, Tian J-H, Yang K-H (2013). Is axillary dissection necessary for breast cancer in old women? A meta-analysis of randomized clinical trials. Asian Pac J Cancer Prev.

[23] Rao R, Euhus D, Mayo HG, Balch C (2013). Axillary node interventions in breast Cancer: a systematic review. JAMA.

[24] Martelli G, Boracchi P, Orenti A, Lozza L, Maugeri I, Vetrella G (2014). Axillary dissection versus no axillary dissection in older T1N0 breast cancer patients: 15-year results of trial and out-trial patients. Eur J Surg Oncol.

[25] Bonneau C, Hequet D, Estevez JP, Pouget N, Rouzier R (2015). Impact of axillary dissection in women with invasive breast cancer who do not fit the Z0011 ACOSOG trial because of three or more metastatic sentinel lymph nodes. Eur J Surg Oncol.

[26] Li CZ, Zhang P, Li RW, Wu CT, Zhang XP, Zhu HC (2015). Axillary lymph node dissection versus sentinel lymph node biopsy alone for early breast cancer with sentinel node metastasis: a meta-analysis. Eur J Surg Oncol.

[27] Giuliano AE, Hunt KK, Ballman KV, Beitsch PD, Whitworth PW, Blumencranz PW (2011). Axillary dissection vs no axillary dissection in women with invasive breast cancer and sentinel node metastasis: a randomized clinical trial. JAMA.

[28] Sávolt Á, Polgár C, Musonda P, Mátrai Z, Rényi-Vámos F, Tóth L (2013). Does the result of completion axillary lymph node dissection influence the recommendation for adjuvant treatment in sentinel lymph node–positive patients?. Clin Breast Cancer.

[29] Sávolt Á, Péley G, Polgár C, Udvarhelyi N, Rubovszky G, Kovács E (2017). Eight-year follow up result of the OTOASOR trial: the optimal treatment of the axilla – surgery or radiotherapy after positive sentinel lymph node biopsy in early-stage breast cancer. Eur J Surg Oncol.

[30] Yao K, Liederbach E, Pesce C, Wang C-H, Winchester DJ (2015). Impact of the American College of Surgeons oncology group Z0011 randomized trial on the number of axillary nodes removed for patients with early-stage breast Cancer. J Am Coll Surg.

[31] de Gregorio A, Widschwendter P, Albrecht S, de Gregorio N, Friedl TWP, Huober J (2018). Axillary surgery in breast Cancer patients treated with breast-conserving surgery at German breast Cancer centers within the last 14 years - comparison of a Unversity center and a community hospital. Geburtsh Frauenheilk.

[32] Hughes KS, Schnaper LA, Bellon JR, Cirrincione CT, Berry DA, McCormick B (2013). Lumpectomy plus tamoxifen with or without irradiation in women age 70 years or older with early breast cancer: long-term follow-up of CALGB 9343. J Clin Oncol.

[33] Kreienberg R, Wöckel A, Wischnewsky M (2018). Highly significant improvement in guideline adherence, relapse-free and overall survival in breast cancer patients when treated at certified breast cancer centres: an evaluation of 8323 patients. The breast.

[34] Wollschläger D, Meng X, Wöckel A, Janni W, Kreienberg R, Blettner M (2017). Comorbidity-dependent adherence to guidelines and survival in breast cancer-is there a role for guideline adherence in comorbid breast cancer patients? A retrospective cohort study with 2137 patients. Breast J.

[35] Black D (2017). Axillary ultrasound: for all, for none, to diagnose positive nodes, or to support avoiding sentinel lymph node biopsy altogether. Ann Surg Oncol.

[36] Sackey H, Magnuson A, Sandelin K, Liljegren G, Bergkvist L, Fülep Z (2014). Arm lymphoedema after axillary surgery in women with invasive breast cancer. Br J Surg.

[37] Jayasinghe UW, Pathmanathan N, Elder E, Boyages J (2015). Prognostic value of the lymph node ratio for lymph-node-positive breast cancer- is it just a denominator problem?. Springerplus..

[38] Wu S-G, Sun J-Y, Zhou J, Li F-Y, Lin Q, Lin H-X (2015). Number of negative lymph nodes is associated with disease-free survival in patients with breast cancer. BMC Cancer..

[39] Wu S-G, Wang Y, Zhou J, Sun J-Y, Li F-Y, Lin H-X (2015). Number of negative lymph nodes should be considered for incorporation into staging for breast cancer. Am J Cancer Res.

[40] Wen J, Ye F, He X, Li S, Huang X, Xiao X (2016). Development and validation of a prognostic nomogram based on the log odds of positive lymph nodes (LODDS) for breast cancer. Oncotarget..

[41] Untch M, Huober J, Jackisch C, Schneeweiss A, Brucker S, Dall P (2017). Initial treatment of patients with primary breast Cancer: evidence, controversies, consensus. Geburtshilfe Frauenheilkd.

[42] Witucki G, Degregorio N, Rempen A, Schwentner L, Bottke D, Janni W (2015). Evaluation of sentinel lymph node dose distribution in 3D conformal radiotherapy techniques in 67 pN0 breast Cancer patients. Int J Breast Cancer..

[43] Zhao M, Liu W-G, Zhang L, Jin Z-N, Li Z, Liu C (2017). Can axillary radiotherapy replace axillary dissection for patients with positive sentinel nodes? A systematic review and meta-analysis. Chronic Dis Transl Med.

[44] Donker M, van Tienhoven G, Straver ME, Meijnen P, van de Velde CJH, Mansel RE (2014). Radiotherapy or surgery of the axilla after a positive sentinel node in breast cancer (EORTC 10981-22023 AMAROS): a randomised, multicentre, open-label, phase 3 non-inferiority trial. Lancet Oncol.

[45] Schwentner L, Wolters R, Koretz K, Wischnewsky MB, Kreienberg R, Rottscholl R (2012). Triple-negative breast cancer: the impact of guideline-adherent adjuvant treatment on survival--a retrospective multi-Centre cohort study. Breast Cancer Res Treat.

[46] Liang S, Hallet J, Simpson JS, Tricco AC, Scheer AS (2017). Omission of axillary staging in elderly patients with early stage breast cancer impacts regional control but not survival: a systematic review and meta-analysis. J Geriatr Oncol..

[47] Joyce DP, Manning A, Carter M, Hill ADK, Kell MR, Barry M (2015). Meta-analysis to determine the clinical impact of axillary lymph node dissection in the treatment of invasive breast cancer. Breast Cancer Res Treat.

[48] Ebner F, Friedl TWP, de Gregorio A, Lato K, Bekes I, Janni W (2018). Seroma in breast surgery: all the surgeons fault?. Arch Gynecol Obstet.

